# Sustaining planetary health in the Anthropocene

**DOI:** 10.7189/jogh.12.03068

**Published:** 2022-11-08

**Authors:** Shilu Tong, Hilary Bambrick

**Affiliations:** 1 Shanghai Children's Medical Center, School of Medicine, Shanghai Jiao Tong University, Shanghai, China; 2 School of Public Health, Institute of Environment and Population Health, Anhui Medical University, Hefei, China; 3 Center for Global Health, School of Public Health, Nanjing Medical University, Nanjing, China; 4 School of Public Health and Social Work, Queensland University of Technology, Brisbane, Australia; 5 National Centre for Epidemiology and Population Health, The Australian National University, Canberra, Australia

The inextricable link between global environmental change and human health has gained attention as we witness increasingly catastrophic weather events and widespread environmental degradation wrought by pollution and biodiversity loss and respond to an ongoing COVID pandemic of zoonotic origins [1,2]. In 2014, Richard Horton et al. called for a collective manifesto to transform public health to planetary health [3], which drew a range of responses [4-6]. Planetary health was defined as the health of human civilisation and the state of the natural systems on which it depends [3]. Soon after that, the 2015 report of the Rockefeller Foundation – Lancet Commission on planetary health clearly indicated that we have been mortgaging the health of future generations to realise short-term economic and development gains in the present [7]. Human civilisation has largely flourished by unsustainably exploiting nature's resources, but now confronts substantial health risks from the degradation of nature's life support systems as a direct result of human activities. Health risks from large-scale environmental changes such as climatic change, ocean acidification, land degradation, water scarcity, overexploitation of fisheries, and biodiversity loss pose serious challenges to global health and are likely to increase over the coming decades because of growing populations, accelerating demand for resources (such as land, food, and energy), and inappropriate use of technology (such as the nuclear armament race). The World Health Organization (WHO) recently estimated that more than 13 million deaths annually occur due to avoidable environmental causes, including climate change, air pollution, and other exposures [8].

Our planetary home – Earth – has existed for 4.6 billion years. Over the last 200 millennia, *Homo sapiens* has flourished from being hunter-gatherers (200 000BP to 8000BP) to agriculturalists (8000BP to 20th century), and then to today’s closely-connected, predominantly high-tech, urban residents [9]. The global population has increased from 1 billion in 1800 to 7.9 billion today and is anticipated to reach approximately 11 billion by the end of this century [10]. The rapid growth of the human population has led to more exploitation of Earth’s resources for food, water, clothing, shelter, and leisure. Significant global changes including climate change, biodiversity loss, urbanization, and ecosystem degradation are accelerating. In 2002, Nobel laureate, atmospheric chemist Paul Crutzen suggested that we may have entered the “Anthropocene epoch,” because humans now play a dominant role in the global environment [11]. Anthropogenic global changes threaten both planetary health and human health. Accumulating evidence suggests that humans have had increasing effects on the planet and the expected negative outcomes and challenges for the planetary systems, which will negatively affect human health [1,2]. We must understand that human health is dependent on planetary health, while the health of the planet does not depend on us at all. We need to shift the relationship in ecological terms from exploitation to symbiosis, where we can be caretakers that provide potential benefits and enjoy the rewards of a healthy planet.

How we sustain planetary health in the Anthropocene is an important issue which needs to be addressed urgently. We propose the following strategies:

First, we need to better understand the major drivers of global changes. Emerging evidence suggests that rapid population growth, over-consumeristic behaviour, and inappropriate use of technology are three key causes of these changes. Therefore, we should develop and implement effective intervention policies and/or measures to slow down these changes and/or prevent further deterioration of planetary health. For instance, maternal and child health and female literacy and education must be improved in poorer nations, while family planning tools (eg, information, pills, and condoms) should be freely available. Furthermore, community education and health promotion are key to halting population overgrowth in economically disadvantaged nations. For rich countries, we should target over-consumeristic behaviour (including changes in food systems) and the inappropriate use of technology, because these factors can waste a considerable amount of natural resources and can damage people’s health and well-being.

**Figure:**
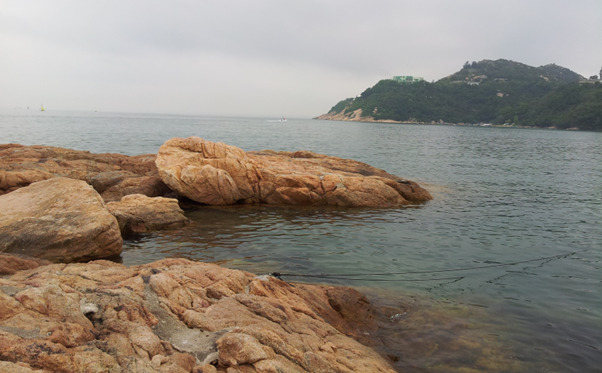
Photo: Human health depends on planetary health. Source: from the authors’ own collection, used with permission.

Second, environmental justice and global equity are imperative to understanding the drivers and implications of the Anthropocene; people living in poverty and instability will be most vulnerable to the impact of global changes, but they contribute little to global crises. We need to reduce inequities within and across societies and educate for empathy so commitments in mitigating and managing environmental changes can be enhanced at all levels (ie, local, regional, national, and global). Planetary health can only be sustained if socioeconomic inequities and environmental injustice are lessened.

Third, we live in an increasingly interconnected and interdependent world and should see ourselves as members of a global village. A strong stewardship concept and a well-coordinated multilateral approach are needed to tackle global crises. There is much to learn on planetary stewardship and successful ways to live more harmoniously within the planetary boundaries, particularly from Indigenous cultures worldwide.

Finally, international partnership and cooperation are key for planetary and human health. Collective actions must be built on cross-sector and inter-generations collaboration because current global crises are unlikely to be resolved by any one sector and/or in one generation. To protect our children and grandchildren from the catastrophic effects of global changes, we need to take collective action now. Planetary and human health cannot be sustained without them.
